# Editorial: Understandings and experiences of colourism: global perspectives

**DOI:** 10.3389/fsoc.2026.1964986

**Published:** 2026-09-30

**Authors:** Aisha Phoenix, Nadia Craddock, Margaret Hunter

**Affiliations:** 1 Centre for Public Policy Research, School of Education, Communication and Society, Faculty of Social Science and Public Policy, King's College London, London, United Kingdom; 2 Centre for Appearance Research, School of Social Sciences, University of the West of England, Bristol, United Kingdom; 3 Department of Sociology, Santa Clara University, Santa Clara, CA, United States

**Keywords:** colorism, education, health, inequality, intersectionality, relationships, skin shade, wellbeing

Prejudice and discrimination in which people are penalized for having darker skin and features not associated with whiteness is known as colorism (Hunter, 2005; Phoenix and Craddock, 2025). Rooted in histories of colonialism, slavery, caste and racialized hierarchies, colorism operates both within and between racialized groups, shaping opportunities and lived experiences across diverse cultural contexts. Colorism has far-reaching consequences for health and wellbeing, education and employment opportunities, interpersonal relationships, and within legal justice systems (Monk, 2019, 2021; Strmic-Pawl et al., 2021), shaping, for instance, who is hired, who is considered desirable as a romantic partner, and who is believed in court. Technological innovations, including the algorithms driving social media platforms and AI-enhanced beauty standards, have only added to the power of global messaging that prioritizes light skin and devalues dark skin. Despite increasing scholarly attention, research has remained disproportionately concentrated on the United States and the experiences of Black Americans, leaving important geographical contexts, populations, and disciplinary perspectives comparatively underexplored. This Research Topic, “Understandings and Experiences of Colourism: Global Perspectives” brings together international scholars whose work advances understanding of how colorism operates across multiple regions, populations, and social institutions. The nine articles in this volume highlight both the global reach of colorism and the diverse ways in which it is experienced, reproduced, and challenged, offering new theoretical, methodological, and empirical insights into an enduring yet under-researched form of inequity. Many articles take an intersectional approach to their investigations of colorism and include studies of colorism and migration, gender, sexuality, and class. For example, Hersch's article investigates the interaction between immigration and colorism, Veak's article explores the relationship between gender and colorism, and Dixon's article examines how class and skin color influence health outcomes. Moreover, the entire volume demonstrates that colorism operates across multiple levels of social life, from everyday interactions to institutions and systems of inequality. We have grouped articles accordingly, though acknowledge that some papers straddle more than one theme.

Three articles in the Research Topic examine how colorism shows up in individuals' everyday lives: online, in relationships, and in family/community settings. First, Majumdar's work qualitatively explores how colorism operates on social media, examining how the video platform TikTok perpetuates, normalizes and commodifies colorism through an examination of British South Asian women's engagement with skincare and beauty content on the platform. Second, another interview study written by two of the guest editors, Phoenix and Craddock, explores how colorist beauty ideals that privilege light skin affect Black and mixed Black-White women's experiences of the heterosexual relationship market. Findings suggest that women with light skin are often desired while those with darker skin are overlooked. In turn, colorist injustices stoke competition between women of different skin shades, with those with light skin holding an advantage in the relationship market. Third, also employing qualitative methods,Veak advances understandings of colorism among Cambodians by examining how family and community shape Khmer women's perceptions of colorism. Veak explores the colorism experiences of Khmer women living in the USA and Cambodia and reports that self-esteem and colorism experiences were shaped by the way in which support from family and cultural connections to Khmer identity interacted with relative skin shade within families and communities. Together, these three studies show colorism operating through ordinary, everyday practices, from engaging with social media to family and peer relationships.

The next three articles in this Research Topic examine associations between colorism and psychological wellbeing, health, and identity. First, Craddock et al. examine associations between experiences of colorism and body image and self-esteem among Black and South Asian adolescents in the UK based on online survey data. One of their major findings is that colorism from people of the same racialized group was a stronger predictor of low self-esteem than colorism from other racialized groups. Second, Dixon's article contributes to the literature on colorism and health by exploring Latin American adults personal and vicarious experiences of skin shade discrimination. She found that witnessing colorism directed at others (i.e., vicarious colorism) was associated with worse self-rated health, while personally experiencing colorism was not. Third, Camacho et al.'s article uses computer mouse tracking to examine how skin shade and prototypicality of Latin Americans, i.e., the extent to which their phenotypical appearance aligns with features associated with Latin Americans, relates to the ways in which they are racialized and stereotyped. These findings complement the qualitative findings of Phoenix and Craddock, by demonstrating that skin shade can shape the processes through which people are racialized and stereotyped. Collectively, these three studies indicate that colorism, like other forms of discrimination, is detrimental to wellbeing and can shape self-perceptions and social belonging.

The final three articles focus on colorism as a system of inequality. Naqvi focuses on the invisibility of colorism in the UK legal system. She argues that colorism is absent from the UK legal framework despite colorism-related incidents being brought to court and suggests that the law is poorly equipped to address racism let alone colorism. Naqvi demonstrates that colorism is neither specifically recognized in the UK hate crime framework, nor the 2010 Equality Act, making legal claims exceptionally difficult. Kelly considers one aspect of colorism in the Caribbean with her article on colorism occurring both between and within racialized groups in Trinidad and Tobago. Drawing on Americas Barometer surveys, she finds that there is a significant association between darker skin and reduced odds of attaining higher levels of education as well as reduced access to household amenities. Hersch's research focuses on an association between skin shade and earnings in the United States. In her analysis of Current Population Survey 2015–2024 data matched to New Immigrant Survey skin color data, she reported that immigrants from countries where people have darker skin experience a significant earnings penalty, even when controlling for age, education, ethnicity, and immigration status. Together, these three articles suggest that colorism operates as a structural force with material consequences across law, education, and the labor market.

Collectively, these nine articles highlight the pervasiveness of colorism and its relevance in different parts of the world. Moreover, they show that colorism operates at different levels within society, enacted through everyday practices and conversations as well as through institutions, and that experiencing colorism is associated with worse wellbeing outcomes. Addressing colorism, many of the authors suggest, requires action at each of these levels. These include: increasing understanding of how it operates; facilitating conversations about colorism; challenging and addressing colorism through educational programs and anti-discrimination policies; improving media representation of people with darker skin and features not associated with whiteness and challenging colorist beauty ideals. The Research Topic also points to important priorities for future scholarship, including longitudinal studies, intervention development and comparative international research. Despite our efforts to include articles from around the world, the Research Topic has no contributions on colorism in sub-Saharan Africa, North Africa, the Middle East or East Asia. It is important that future collections foreground research in these regions to further understandings of how colorism operates.
